# Cryo-EM structure of the *Pseudomonas aeruginosa* MexY multidrug efflux pump

**DOI:** 10.1128/mbio.03826-24

**Published:** 2025-03-05

**Authors:** William D. Gregor, Rakesh Maharjan, Zhemin Zhang, Lucius Chiaraviglio, Nithya Sastry, Meng Cui, James E. Kirby, Edward W. Yu

**Affiliations:** 1 Department of Pharmacology, Case Western Reserve University School of Medicine, Cleveland, Ohio, USA; 2 Department of Pathology, Beth Israel Deaconess Medical Center, Boston, Massachusetts, USA; 3 Department of Pharmaceutical Sciences, Northeastern University School of Pharmacy, Boston, Massachusetts, USA; 4 Harvard Medical School, Boston, Massachusetts, USA; Dana-Farber Cancer Institute, Boston, Massachusetts, USA

**Keywords:** multidrug resistance, multidrug efflux pump, MexY, *Pseudomonas aeruginosa*, cryo-EM

## Abstract

*Pseudomonas aeruginosa*, a Gram-negative pathogen, has emerged as one of the most highly antibiotic-resistant bacteria worldwide and subsequently has become a leading cause of healthcare-associated, life-threatening infections. *P. aeruginosa* multidrug efflux Y (MexY) is an efflux pump that belongs to the resistance-nodulation-cell division (RND) superfamily. It is a major determinant for resistance to aminoglycosides in this opportunistic pathogen. However, the detailed molecular mechanisms involved in aminoglycoside recognition and extrusion by MexY have not been elucidated. Here, we report the cryo-electron microscopy structure of MexY to a resolution of 3.63 Å. The structure directly indicates two plausible pathways for drug export. It also suggests that MexY is capable of picking up antibiotics via the ceiling of the central cavity formed by the MexY trimer. Molecular dynamics simulations depict that MexY is able to use a tunnel connecting the central cavity to the funnel of the trimer to export its substrates.

**IMPORTANCE:**

Here, we report the cryo-electron microscopy structure of the MexY multidrug efflux pump, posing the possibility that this pump is capable of capturing antibiotics from both the central cavity and the periplasmic cleft of the pump. The results indicate that MexY may utilize charged residues to bind and export drugs, mediating resistance to these antibiotics.

## INTRODUCTION

*Pseudomonas aeruginosa* is a highly drug-resistant Gram-negative bacterium that belongs to the ESKAPE (*Enterococcus faecium*, *Staphylococcus aureus*, *Klebsiella pneumoniae*, *Acinetobacter baumannii*, *Pseudomonas aeruginosa*, and *Enterobacter* species) group of pathogens. These ESKAPE pathogens are the primary cause of nosocomial infections and present one of the greatest challenges to modern medicine as most of these bacteria have evolved to become resistant to multiple antimicrobials (1, 2). *P. aeruginosa* is a leading cause of healthcare-associated, life-threatening infections, including pneumonia, septicemia, and infections after surgery. Infections in critically ill and immunocompromised patients, especially those with cystic fibrosis, are especially problematic and often cause significant morbidity (3).

Multidrug efflux is a powerful antibiotic resistance mechanism and a primaryr cause of failure of drug-based treatments of infectious diseases (4). A major contributor to the intrinsic and acquired bacterial resistance of microbials are multidrug efflux pumps of the resistance-nodulation-cell division (RND) superfamily (5). An RND multidrug efflux pump is an inner membrane protein that interacts with a periplasmic membrane fusion protein and an outer membrane channel protein to assemble as a tripartite efflux complex to extrude antimicrobials directly out of cells (6). One such RND efflux pump, *P. aeruginosa* MexY, is particularly noteworthy because of its unusual role in mediating resistance to aminoglycoside drugs, including streptomycin, paromomycin, neomycin, and amikacin (7). MexY is also able to confer resistance to a range of other antimicrobial agents, such as fluoroquinolones, macrolides, tetracyclines, spectinomycin, chloramphenicol, and cefepime (7, 8). This efflux pump is an inner membrane protein that works in conjunction with the MexX periplasmic membrane fusion protein and the OprM outer membrane channel to form the MexXY-OprM tripartite efflux system. MexY is a substrate/proton antiporter. It constitutes substrate-binding sites and utilizes the proton motive force (PMF) to export these antimicrobials out of the cell (8).

To date, the only structural information available for any aminoglycoside-specific RND efflux pump available is the *Escherichia coli* AcrD membrane protein (9). This membrane protein depends upon charged residues to mediate resistance to aminoglycoside drugs, where this pump is able to pick up aminoglycosides from the central cavity of the AcrD trimer and eliminate them from the cell via a drug export tunnel surrounded with charged amino acids (9). To further elucidate the molecular mechanisms of aminoglycoside-specific RND efflux pumps, here, we report the cryo-electron microscopy (cryo-EM) structure of the *P. aeruginosa* MexY aminoglycoside efflux pump to a resolution of 3.63 Å. Combined with molecular dynamics (MD) and targeted MD simulations, our data provide a detailed pathway for substrate transport via the MexY membrane protein.

## RESULTS AND DISCUSSION

### Structures of MexY

**Fig 1:**
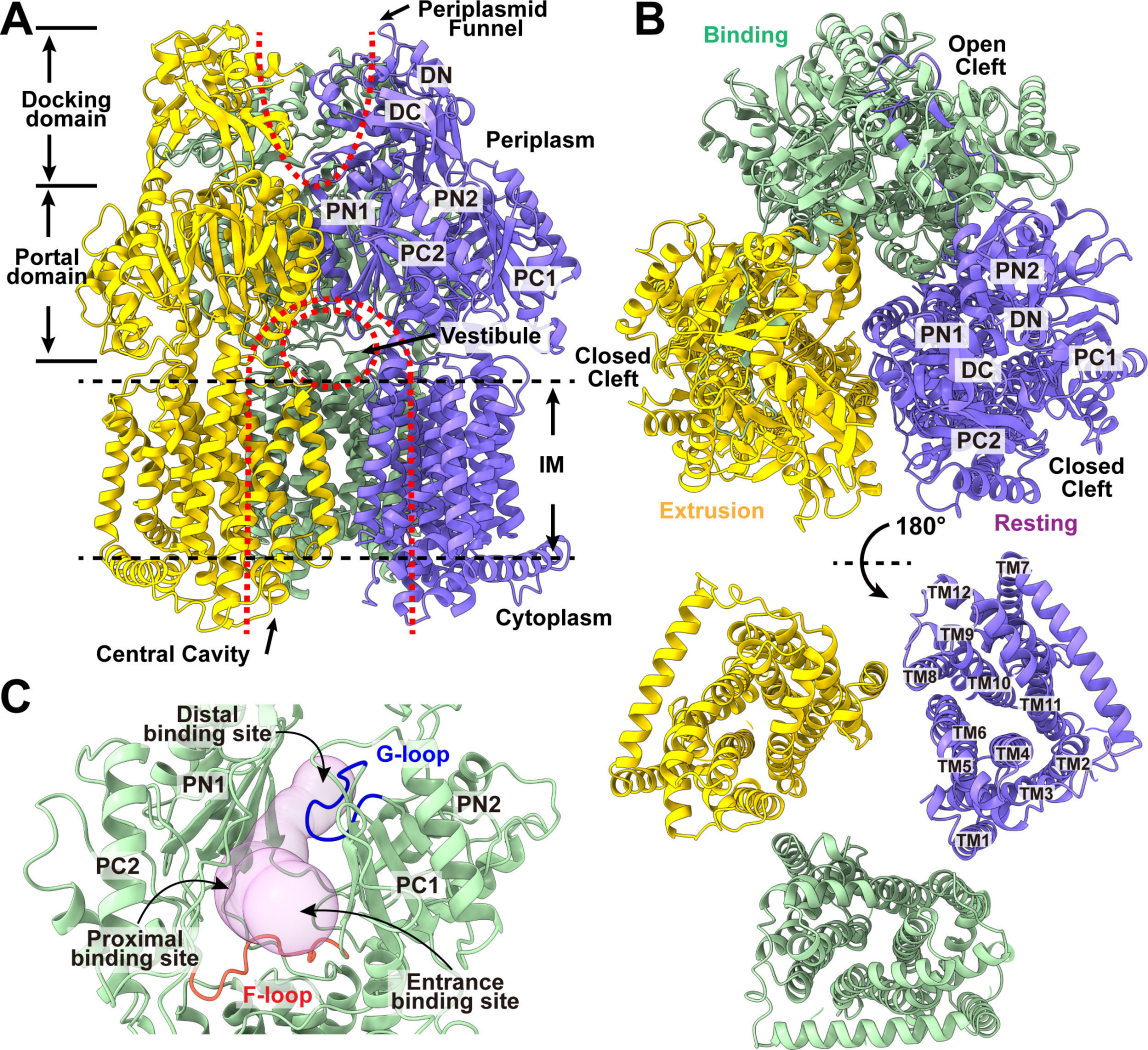
Cryo-EM structure of trimeric MexY. Ribbon diagrams of the structure of the (**A**) side view (viewed in the membrane plane), (**B**) top view (viewed from the extracellular space), and bottom view (viewed from the cytoplasm) of the MexY trimer. In panels **A** and **B**, the “binding,” “resting,” and “extrusion” protomers are colored green, blue, and yellow, respectively. Each protomer of MexY contains 12 TM helices (TM1–TM12) and six periplasmic subdomains (PN1, PN2, PC1, PC2, DN, and DC). The locations of the central cavity, vestibule, and funnel are labeled. (**C**) The periplasmic cavity of MexY. This large, spacious cavity (pink) inside the periplasmic cleft between subdomains PC1 and PC2 forms the entrance, proximal, and distal binding sites. The F-loop (red) connects the entrance and proximal sites, whereas the G-loop (blue) separates the proximal and distal sites into two compartments.

The cryo-EM structure of MexY reveals that this multidrug efflux pump forms a homotrimer and adopts the overall fold of hydrophobe-amphiphile efflux (HAE)-RND membrane proteins (10–15) (Fig. S1; Table S1A). Each MexY protomer possesses a transmembrane (TM) domain composed of 12 TM helices (TM1–TM12) and a periplasmic domain consisting of six subdomains (PN1, PN2, PC1, PC2, DN, and DC) (Fig. 1A and B). Within the TM domain, the TMs, particularly TM4 and TM10, are responsible for generating a proton-relay network to transfer protons from the periplasm to the cytoplasm and producing the PMF. In the periplasmic domain, subdomains PC1 and PC2 form a periplasmic cleft that provides sites for substrate binding (Fig. 1C). Coupled with the PMF, this cleft can be opened to create an entrance and allow substrates to enter the pump for extrusion from the periplasmic space.

Within the HAE-RND efflux pump, there resides a large periplasmic cavity. This cavity is compartmentalized into several multidrug binding sites, including the entrance, proximal, and distal sites (16). This cleft also contains two functional loops: the F-loop (flexible loop) and the G-loop (gate loop) (Fig. 1C). The F-loop connects the entrance and proximal sites, whereas the G-loop sectionalizes the proximal and distal sites into two compartments. Presumably, a drug molecule entering the entrance site will be led by the F-loop to shuttle it to the proximal drug-binding site, where it will transit across the G-loop and be delivered to the distal site before final drug extrusion (Fig. 1C). Indeed, a cryo-EM structure of *A. baumannii* AdeB bound by three ethidium bromide (Et) molecules within a single protomer of the AdeB trimer has shown that the three bound Et molecules line along this path, providing a mechanism for drug recognition and extrusion (17).

**Fig 2:**
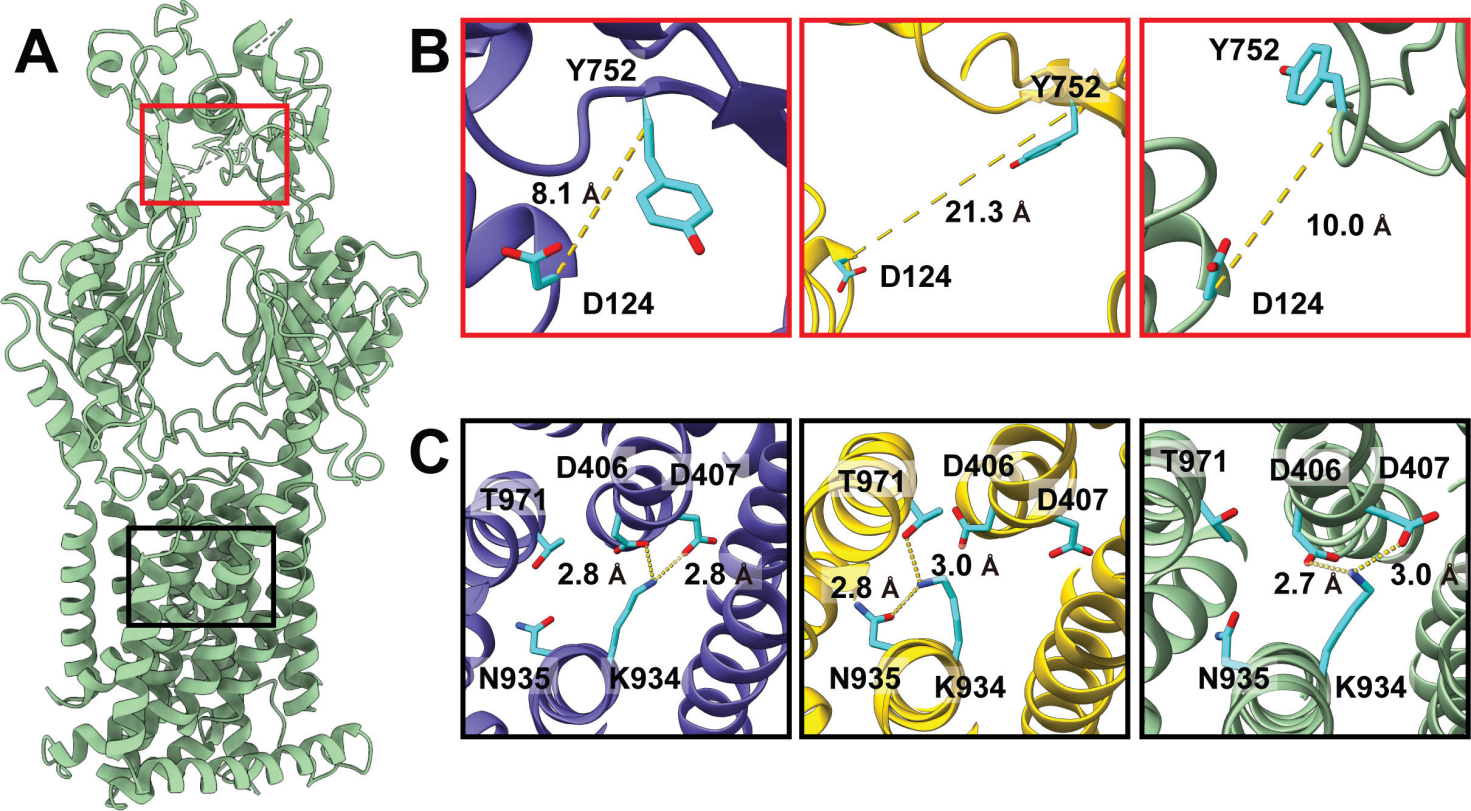
Measurements of the exit sites and proton-relay networks of MexY. (**A**) Ribbon diagram of a MexY protomer of trimeric MexY showing the locations of the exit site (red box) and proton-relay network (black box). (**B**) Distance between the Cα atoms of D124 and Y752, which form the exit site for drug, for the three MexY protomers in the trimeric MexY structure. (**C**) Conformational states of the proton-relay network of the three MexY protomers in the trimeric MexY structure.

Similar to the assembly of many other HAE-RND pumps, the cryo-EM structure of MexY displays an asymmetric, homotrimeric architecture with the pseudo-threefold axis positioned perpendicular to the membrane surface. The conformational states of the three MexY protomers assembled to form this trimer can be assigned as “binding,” “resting,” and “extrusion” states (Fig. 2A through C; Table S1B), where the periplasmic cleft established between PC1 and PC2 of the "binding” conformer is open, but the periplasmic clefts in both the "extrusion" and "resting" conformers of this pump are closed (Fig. 1A and B). This same combination of conformational states within a trimer has been observed in *E. coli* AcrD (9), *A. baumannii* AdeB (17), and *Campylobacter jejuni* CmeB (14).

**Fig 3:**
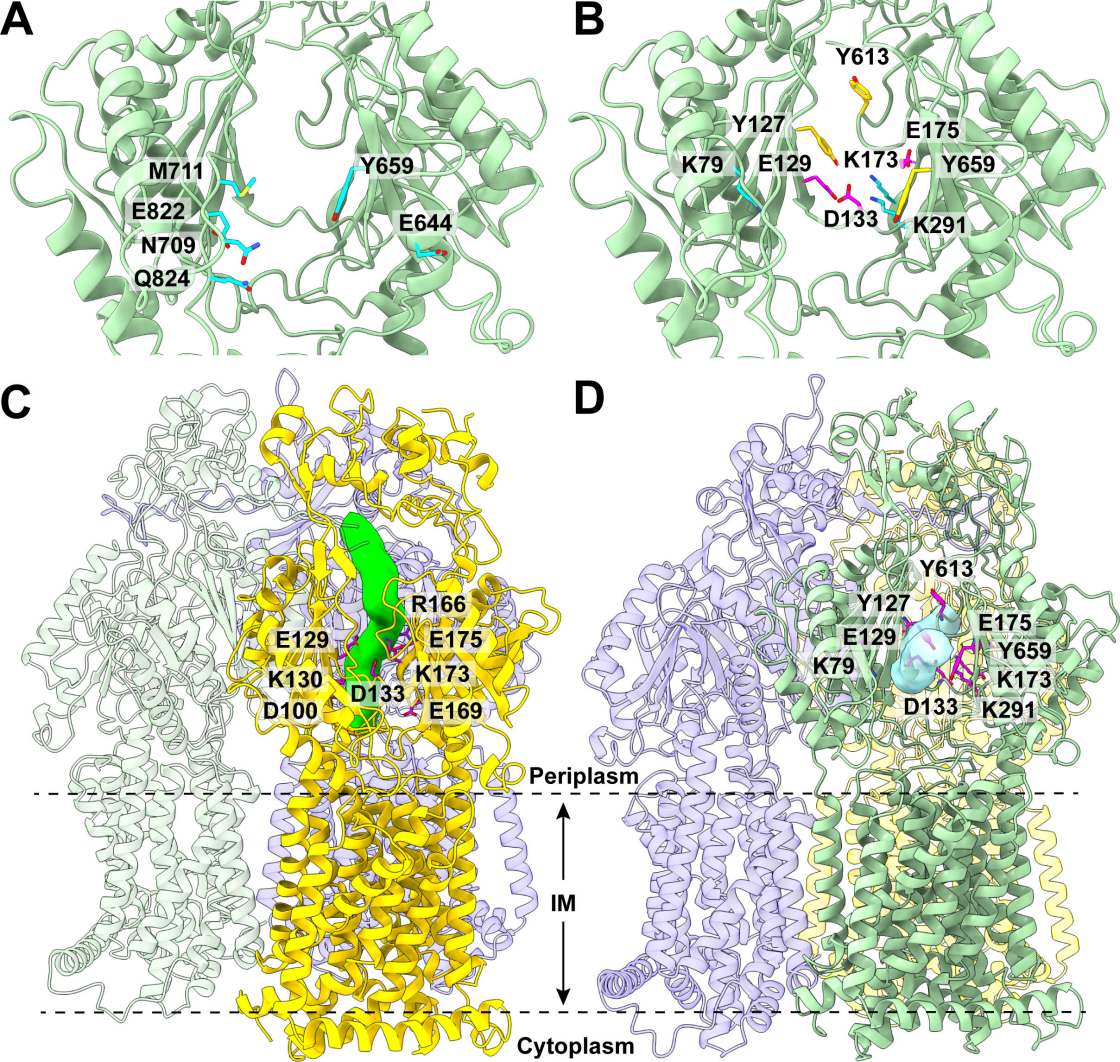
The periplasmic multidrug binding sites. (**A**) The entrance binding site. This site is surrounded by residues E644, Y659, N709, M711, E822, and Q824, which are colored cyan. (**B**) The proximal and distal binding sites. The large periplasmic cavity constituting the proximal and distal binding sites is surrounded with several anionic (magenta; E129, D133, and E175) and aromatic (yellow; Y127, Y613, and Y659) residues. It also contains several lysines (cyan), including K79, K173, and K291. (**C**) The extrusion tunnel of MexY. This extrusion tunnel (green) is surrounded by charged residues, such as D100, E129, K130, D133, R166, E168, K173, and E175. (**D**) The binding tunnel of MexY. This binding tunnel is colored cyan. The anionic (E129, D133, and E175), cationic (K79, K173, and K291), and aromatic (Y127, Y613, and Y659) residues are included, where these residues are colored magenta.

The entrance site of the MexY periplasmic cleft is surrounded by anionic or polar residues E644, Y659, N709, M711, E822, and Q824 (Fig. 3A). These residues likely play a role in substrate specificity and selectivity. It should be noted that these MexY residues are not conserved with other HAE-RND pumps. Interestingly, M711 of *P. aeruginosa* MexY corresponds to R710 of *C. jejuni* RE-CmeB (18), R711 of *C. jejuni* CmeB (14), R714 of *N. gonorrhoeae* MtrD (13, 19, 20), R715 of *E. coli* AcrD (9), R716 of *P. aeruginosa* MexB (12), R716 of *K. pneuominae* AcrB (21), R717 of *E. coli* AcrB (10, 11, 16), and R718 of *A. baumannii* AdeJ (22, 23), where all of these corresponding arginine residues are conserved among these pumps. These conserved arginines have also been shown to be important for substrate specificity (24–26). Therefore, the substrate selectivity and specificity of MexY are expected to be quite distinct.

As mentioned above, the MexY pump constitutes a large, spacious substrate-binding cavity deep inside the periplasmic cleft. This cavity, compartmentalized into the proximal and distal drug-binding sites, is surrounded with several anionic residues, such as E129, D133, and E175, and aromatic residues, including Y127, Y613, and Y659 (Fig. 3B). Based on the structural information, these anionic and aromatic residues may be critically important for substrate recognition, where MexY may prefer to bind positively charged drugs. Interestingly, it has been observed that mutations in D133 and Y613 significantly compromised aminoglycoside resistance mediated by the MexY pump (7), highlighting the critical functional roles of these two amino acids.

In addition, the periplasmic binding cavity contains several lysines, including K79, K173, and K291 (Fig. 3B). These cationic residues may also be important for the function of MexY. Indeed, a substitution at position K79 was found to enhance resistance to aminoglycosides (7).

*P. aeruginosa* MexY and MexB share 50% protein sequence identity. Even though their substrate specificities are quite different, there is some overlap between the two exporters. MexB is capable of extruding fluoroquinolones, macrolides, tetracyclines, chloramphenicol, novobiocin, and most β-lactams, mediating resistance to these antimicrobials (8). However, MexB cannot recognize the aminoglycoside class of drugs. The periplasmic entrance, proximal, and distal drug-binding sites of MexB are surrounded by several positively and negatively charged amino acids, such as K134, D174, R620, and R649 (Fig. S2). In particular, the crystal structure of lauryl maltose neopentyl glycol (LMNG)-bound MexB indicates that K134 and R620 are critical for substrate binding (27). Strikingly, the corresponding charged residues in MexY are D133, K173, D615, and E644 (Fig. S2). The charges of these residues are found to switch from positive to negative or vice versa when compared with those in MexB. This observation indeed may explain the difference in substrate specificity of these two efflux pumps.

### Extrusion tunnel of MexY

Previously, we solved cryo-EM structures of *E. coli* AcrD, providing the first structural information of any RND-type aminoglycoside efflux pump. Importantly, the structures allowed us to understand how this pump binds and recognizes aminoglycosides (9). In addition to picking up drug molecules from the open periplasmic cleft of the “binding” protomer, it was observed that the “extrusion” protomer of AcrD utilizes negatively charged amino acids to anchor a gentamicin molecule at the ceiling of the central cavity formed by the AcrD trimer. A tunnel connecting the aminoglycoside binding site at the ceiling of the central cavity to the substrate exit site at the trimer's periplasmic funnel is found in the “extrusion” protomer and thus is capable of shuttling gentamicin across the entire periplasmic domain of the AcrD pump. This tunnel is surrounded by charged amino acids that are critical for recognizing and extruding aminoglycosides (9).

Within the “extrusion” protomer of MexY, the cryo-EM structure indicates a similar tunnel, where it vertically connects the opening situated at the ceiling of the central cavity up to the exit site located at the bottom of the funnel region of the MexY trimer. Like the extrusion tunnel of AcrD, this MexY extrusion tunnel is surrounded with charged residues such as D100, E129, K130, D133, R166, E169, K173, and E175 (Fig. 3C). Based on this observation, the MexY pump likely uses these charged residues with a similar mechanism utilized by AcrD to remove toxic compounds out of the cell. In addition to the extrusion tunnel, we noticed a formation of a horizontal tunnel at the “binding” protomer of MexY (Fig. 3D). This binding tunnel allows for the interior of the periplasmic domain of the “binding” protomer to be exposed to solvent via the entrance site of the periplasmic cleft created between subdomains PC1 and PC2. As observed in the AdeB pump bound with Et molecules (17), this binding tunnel creates a path and directly connects different substrate-binding sites within the periplasmic domain.

### Docking calculations

**Fig 4:**
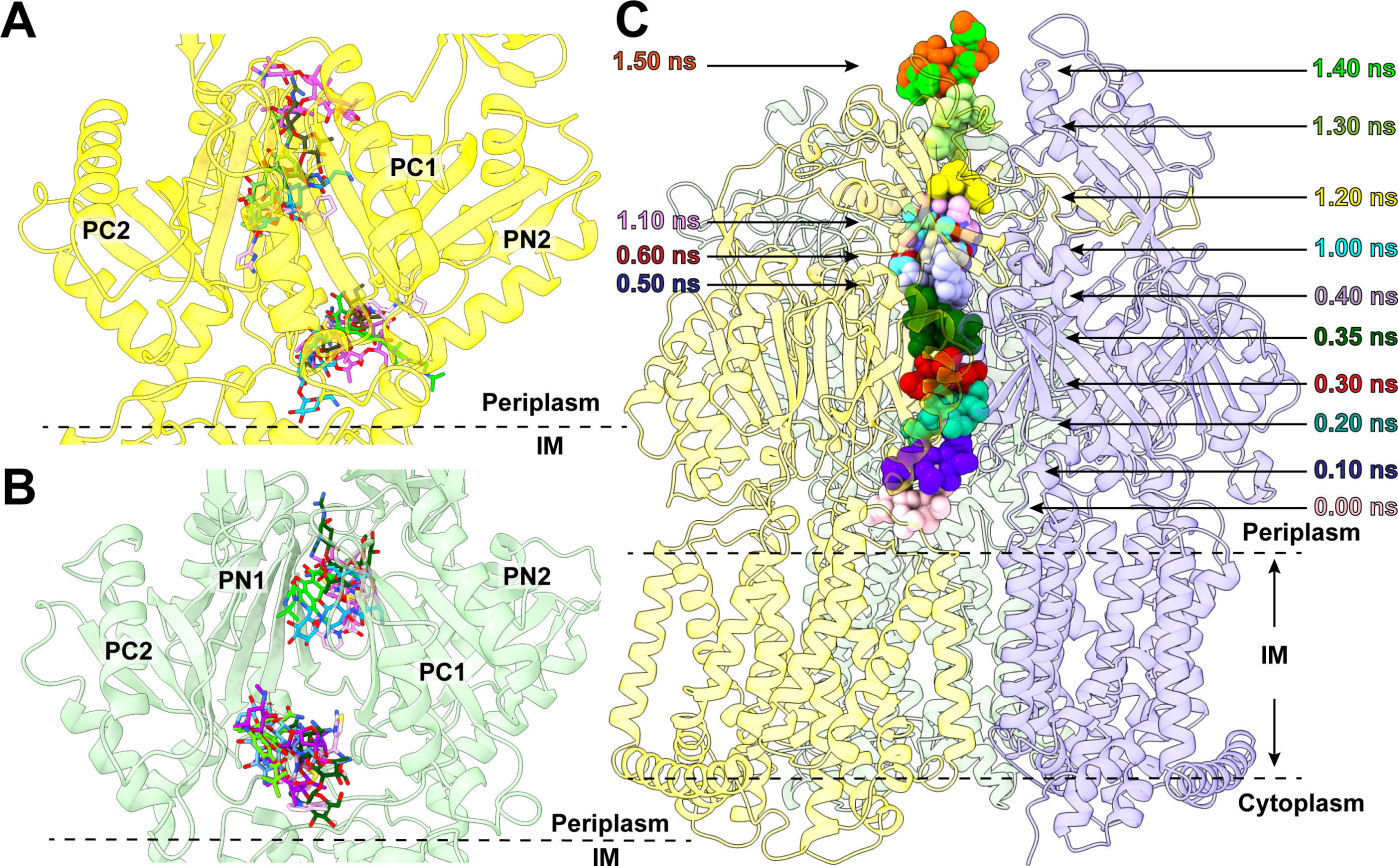
Docking of antibiotics and targeted MD simulations. (**A**) Predicted locations of bound antibiotics in the “extrusion” protomer of MexY. The drugs are found to bind within a pocket located at the central cavity's ceiling and a cavity near the bottom of the funnel at the distal drug-binding site. (**B**) Predicted locations of bound antibiotics in the “binding” protomer of MexY. The drugs are observed to cluster within the entrance drug-binding and distal drug-binding sites. In panels **A** and **B**, bound Akn, Cef, Ery, Str, and T1c are colored blue, light pink, magenta, dark green, and green, respectively. (**C**) Targeted MD simulations of the MexY pump. The calculations depict snapshots (0, 0.10, 0.20, 0.30, 0.35, 0.40, 1.00, 1.20, 1.30, and 1.40 ns) of Str shuttling via the periplasmic tunnel of MexY.

To further elucidate how MexY binds drugs, we used the program AutoDock Vina (28) to predict potential drug-binding modes and drug-pump interactions within the MexY trimer. A panel of antibiotics, including amikacin (Akn), cefpirome (Cef), erythromycin (Ery), streptomycin (Str), and tigecycline (T1c), was used as MexY is capable of mediating resistance to these drugs (7, 8). Within the MexY trimer, it was found that all of these drugs are able to bind within the periplasmic domains of the “extrusion” and “binding” protomers (Fig. 4). In the “extrusion” protomer, these drugs are found to bind in two locations: within a pocket located at the central cavity's ceiling and in a cavity near the bottom of the funnel of the distal drug-binding site (Fig. 4A). In the “binding” protomer, these drugs are observed to cluster within the entrance drug-binding and distal drug-binding sites (Fig. 4B). The docking results indeed highlight two plausible pathways for drug extrusion, where a drug molecule can enter the pump via the entrance located at the ceiling of the central cavity and/or the periplasmic cleft between subdomains PC1 and PC2.

### Computational simulations

Similar to AcrD, the cryo-EM structure of MexY indicates that a tunnel is found at the periplasmic domain of the “extrusion” protomer, where this tunnel spans the entire periplasmic region, from the ceiling of the central cavity up to the bottom of the periplasmic funnel (Fig. 3C). The wall of this tunnel is surrounded by charged residues, including D100, E129, K130, D133, R166, E169, K173, and E175. The narrowest region of the tunnel is restricted by residues E129, K130, and D133. Several of these charged residues, such as K130, D133, and R166, are conserved with those of AcrD. These corresponding AcrD residues are K131, D134, and R167, where they play an important role for AcrD to transport aminoglycosides out of the cell (9). Therefore, it is expected that these MexY-charged residues may be critical for the function of the efflux pump. To elucidate the mechanism of aminoglycoside transport, we performed MD (Fig. S3A through C) and targeted MD (Fig. 4C) simulations on the MexY efflux pump in an explicit lipid bilayer and water environment using Amber (29, 30) and NAMD (31).

#### Dynamics of the MexY efflux pump

Principle component analysis of the MexY trimer indicates that the first and second eigenvectors (Fig. S3; Movies S1 and S2), which depict the two most important motions extracted from the MD simulation trajectory, correspond to a rigid-body rotational movement of the periplasmic domain of each protomer with respect to each other. The hinge of this movement is located between the periplasmic docking and portal domains of the MexY trimer. A similar rigid-body motion has been observed in the AdeB trimer; however, the AdeB rotational movement is more pronounced in that this motion can be transmitted to the TM region to dissociate and associate the trimeric oligomerization of the TM domain (17). In addition, the root mean square fluctuation of residues comprising the DN and DC subdomains is significantly more dramatic (Fig. S3C), indicating that the DC and DN subdomains of the MexY trimer are quite flexible. We postulate that the conformational flexibility of the docking subdomain along with this rigid-body rotational movement may be important for the MexY pump to recruit the MexX membrane fusion protein and the OprM outer membrane channel to form the MexXY-OprM tripartite efflux complex for drug extrusion.

#### Aminoglycoside transport pathway

We believe that MexY is capable of picking up aminoglycoside drugs via both the periplasmic cleft and the central cavity of the pump. Tthe cryo-EM structure of an *A. baumannii* AdeB protomer bound by three Et molecules within the binding tunnel has clearly demonstrated that the three Et molecules line the path formed by the binding tunnel within the periplasmic cleft and that targeted MD simulations have suggested that this binding tunnel can export drugs within 15 ns (17), we therefore focused on elucidating the feasibility of drug export via the tunnel connecting the ceiling of the central cavity and the bottom of the funnel of the MexY pump. We chose the Str aminoglycoside drug for these simulations as MexY is capable of mediating a high level of resistance to this drug (7). Targeted MD simulations indeed allow us to observe that the Str molecule is able to follow the path of the tunnel identified from the cryo-EM structure of MexY and shuttle from the central cavity to the funnel region, across the periplasmic domain of the pump (Fig. 4C; Movie S3). Our data show that Str can arrive at the funnel within 1 ns. In addition, targeted MD simulations suggest that the charged amino acids, particularly D100, E129, K130, and D133, surrounding the wall of this tunnel participate in contacting Str to facilitate the transport of this drug molecule.

#### Putative proton transfer pathway

The HAE-RND efflux pumps are PMF-dependent that function via a drug/proton antiport mechanism (5). Coupled with the export of drugs, protons are imported to energize the process of drug extrusion. Within the TM region of MexY, several conserved charged and polar residues including D406, D407, K934, N935, and T971 are observed. Their corresponding residues were perceived to create important proton-relay networks in other HAE-RND pumps for proton transfer and energy coupling (9, 14, 15, 18, 19, 21, 22, 32). Therefore, it is expected that these MexY residues create a proton-relay network at the TM region and are critical for the function and energy coupling of the pump. In addition to these conserved residues, several charged residues, such as E413, R417, E941, D945, and R964, seemingly constitute a funnel-like shape to face the cytoplasm. These charged residues may also be involved in forming the proton-relay network or a water tunnel to facilitate the transfer of protons.

We used the program Caver (https//www.caver.cz/) to calculate possible tunnels formed within the TM domain of each MexY protomer of the trimer. In each protomer, a funnel-shaped tunnel spanning the cytoplasmic membrane surface up to residues D406, D407, and K934, which form a conserved triad within the proton-relay network, is created between TM4 and TM10 (Fig. S4A). The cluster of charged amino acids (E413, R417, E941, D945, and R964), located below the conserved, charged triad, is indeed found to surround the wall of this funnel-shaped tunnel. This tunnel allows for the interior of the TM domain to be exposed to water. Based on the architecture of the tunnel, it is expected that water is freely permeable from the cytoplasmic side to at least the constriction site formed by the charged triad D406, D407, and K934.

To continue to elucidate the putative proton transfer pathway of MexY, we performed 1 μs MD simulations on the extrusion protomer of this membrane protein. We identified that a water molecule is able to enter the TM domain near the charged residue D918 located at the periplasmic surface of the cytoplasmic membrane (Fig. S4B and C). This water molecule passes near residue T998 and then residue T927. It eventually arrives at a cluster of residues that include the conserved charged and polar residues D406, D407, K934, N935, and T971 and three additional polar residues T445, S972, and T1008 (Fig. S4B and C). This cluster of residues is located right above another cluster of charged amino acids formed by E413, R417, E941, D945, and R964, which create a water tunnel and allow water molecules to enter the TM domain of MexY from the cytoplasmic surface. Subsequently, the water molecule can be easily migrated into the water channel for facilitating proton transfer (Fig. S4C). All of these water-accessible residues likely play a crucial role in forming the proton-relay network for energy coupling in the pump.

## MATERIALS AND METHODS

### Expression and purification of MexY

The *P. aeruginosa* MexY multidrug efflux pump was cloned into the pET15bΩ*mexY* expression vector in frame with a 6×His tag at the C-terminus. The plasmid was transformed into *E. coli* BL21(DE3)*ΔacrB* cells, which harbor a deletion in the chromosomal *acrB* gene, for overproduction of the MexY membrane protein. Cells were grown in 6 L of a Luria-Bertani medium supplemented with 100 µg/mL ampicillin at 37°C. When the OD_600_ reached 0.5, the expression of MexY was induced with 0.2 mM isopropyl-β-D-thiogalactopyranoside. Cells were then harvested within 4 h of induction. The collected bacterial cells were resuspended in low salt buffer (100 mM sodium phosphate [pH 7.2], 10% glycerol, 1 mM ethylenediaminetetraacetic acid [EDTA], and 1 mM phenylmethanesulfonyl fluoride [PMSF]) and disrupted with a French pressure cell. The membrane fraction was collected and washed twice with high salt buffer (20 mM sodium phosphate [pH 7.2], 2 M KCl, 10% glycerol, 1 mM EDTA, and 1 mM PMSF) and once with final buffer (20 mM HEPES-NaOH buffer [pH 7.5] and 1 mM PMSF). The membrane protein was then solubilized in 2% (wt/vol) *n*-dodecyl-β-D-maltoside (DDM). Insoluble material was removed by ultracentrifugation at 100,000 × g. The extracted protein was then purified with a Ni^2+^-affinity column. The purified protein was dialyzed against 20 mM Na-HEPES (pH 7.5) and concentrated to 7 mg/mL (60 µM) in a buffer containing 20 mM Na-HEPES (pH 7.5) and 0.05% DDM.

### Nanodisc preparation

To assemble MexY into nanodiscs, we incubated a mixture containing 20 µM MexY, 45 µM membrane scaffold protein 1E3D1 (MSP1E3D1) (Sigma-Aldrich), and 930 µM *E. coli* total extract lipid at room temperature for 15 min. We added 0.8 mg/mL of pre-washed Bio-beads (Bio-Rad) to remove the DDM detergent. The resultant mixture was incubated for 1 hour on ice followed by overnight incubation at 4°C. The protein-nanodisc solution was filtered through 0.22 µm nitrocellulose-filter tubes to remove the Bio-beads. The filtered protein-nanodisc solution was further purified using a Superose 6 column (GE Healthcare) equilibrated with 20 mM Tris-HCl (pH 7.5) and 100 mM NaCl. Fractions corresponding to the size of the trimeric MexY-nanodisc complex were collected for cryo-EM sample preparation. 

### Cryo-EM sample preparation

For imaging MexY, we directly applied a 10 µM MexY-nanodisc sample to glow-discharged holey carbon grids (Quantifoil Cu R1.2/1.3, 300 mesh), blotted it for 18 s and then plunge-frozen it in liquid ethane using a Vitrobot (Thermo Fisher). The grids were then transferred into cartridges prior to data collection.

### Data collection

The images of MexY were collected in super-resolution mode at 81 K magnification on a Titan Krios equipped with a K3 direct electron detector (Gatan). The physical pixel size was 1.07 Å/pix (super-resolution of 0.535 Å/pix). Each micrograph was exposed to a total dose of 37.6 e^−^/Å^2^ for 5  s, and 38 frames were captured using SerialEM (33).

### Data processing

The super-resolution image stack was aligned and binned by two using patch motion. The contrast transfer function (CTF) was estimated using patch CTF in cryoSPARC (34). A procedure for blob picker followed by two-dimensional (2D) classification was applied to generate templates for automated template picking. Initially, 1,124,205 particles were selected after autopicking in cryoSPARC (34). Several iterative rounds of 2D classifications followed by *ab initio* and heterogeneous three-dimensional (3D) classifications were performed to remove false picks and classes with unclear features, ice contamination, or carbon. The 3D classification analysis was then employed. A single round of non-uniform refinement followed by local refinement with non-uniform sampling resulted in a 3.63 Å resolution cryo-EM map based on the gold standard Fourier shell correlation (FSC 0.143) (Fig. S1).

### Model building and refinement

Model building of trimeric MexY was based on the cryo-EM map. The trimeric AcrD aminoglycoside efflux pump structure (PDB ID: 8F3E) (9) was used as a template and fitted into the corresponding density maps using Chimera (35). The subsequent model rebuilding was performed using Coot (36). Structural refinements were performed using the phenix.real_space_refine program (37) from the PHENIX suite (38). The final atomic model was evaluated using MolProbity (39). The statistics associated with data collection, 3D reconstruction, and model refinement are included in Table S1A.

### Molecular docking

The program AutoDock Vina (28) was used to predict the binding modes of amikacin (Akn), cefpirome (Cef), erythromycin (Ery), streptomycin (Str), and tigecycline (T1c). The trimeric MexY structure was used for dockings. The protein was set as a rigid structure, whereas the conformation of each antibiotic molecule was optimized via all modeling and docking procedures. For each antibiotic, the results were ranked on the basis of predicted free binding energy, where the one with the highest binding affinity was recorded (Table S2).

### MD simulations

The protonation states of the titratable residues of the MexY pump were determined using the H++ server (http://newbiophysics.cs.vt.edu/H++/). The trimeric MexY cryo-EM structure was immersed in an explicit lipid bilayer consisting of POPC, POPE, POPS, and Chl with a molecular ratio of 25:5:5:1 using the CHARMM-GUI Membrane Builder webserver (http://www.charmm-gui.org/?doc=input/membrane). A water box with dimensions of 142.5 Å × 142.5 Å × 173.8 Å was employed. We added 150 mM NaCl and extra neutralizing counter ions for these simulations. The total number of atoms was 333,722. The Antechamber module of AmberTools was employed to generate parameters for Gen using the general AMBER force field (29, 30). The partial charges of Str were calculated using *ab initio* quantum chemistry at the HF/6-31G* level (GAUSSIAN 16 program) (Gaussian Inc., Wallingford). The RESP charge-fitting scheme was used to calculate partial charges on the atoms (*45*). The tleap program was used to generate parameter and coordinate files using the ff14SB and Lipid17 force field for both the protein and lipids. The PMEMD.CUDA program implemented in AMBER18 (AMBER 2018, University of California, San Francisco) was used to conduct MD simulations. The simulations were performed with periodic boundary conditions to produce isothermal-isobaric ensembles. Long-range electrostatics were calculated using the particle mesh Ewald method (40) with a 10 Å cutoff. Prior to the calculations, energy minimization of these systems was carried out. Subsequently, the systems were heated from 0 K to 303 K using Langevin dynamics with a collision frequency of 1 ps^−1^. During heating, the MexY pump was position-restrained using an initial constant force of 500 kcal/mol/Å^2^ and weakened to 10 kcal/mol/Å^2^, allowing lipid and water molecules to move freely. Then, the systems went through 5 ns equilibrium MD simulations. Finally, a total of 1 μs production MD simulations were conducted. During simulations, the coordinates were saved every 100 ps for analysis. The system was well equilibrated after 100 ns simulations according to root mean square deviations of the protein Cα atoms (Fig. S3A). GROMCAS analysis tools were used for the MD simulation trajectory analysis (41).

### Target MD simulations

Target MD (TMD) simulations were performed, based on the MD equilibrated coordinates (1 µs), using the NAMD v2.13 program (31) with the same AMBER force field parameters as described above. Str was docked into the trimeric MexY structure using the Glide program (Schrödinger LLC). In the simulations, we selected the heavy atoms of the Str ligand bound at the Str binding site to be guided toward the target position of the ligand by the application of steering forces. The root mean square (RMS) distance between the current coordinates and the target structure was calculated at each timestep. The force on each selected atom was given by a gradient of potential as a function of the RMS values. Then, TMD simulation was performed for 2 ns on the MexY-Str system. A value of 500 kcal/mol/Å^2^ was used as an elastic constant for TMD forces during the simulations. The coordinates were saved every 1 ps for analysis.

## Data Availability

Atomic coordinate and EM map for MexY have been deposited with PDB accession code 9E9F and EMDB accession code EMD-47796.

## SUPPLEMENTAL MATERIAL

Fig. S1 mbio.03826-24-s0001.tiff Cryo-EM structure of the MexY efflux pump.

Fig. S2 mbio.03826-24-s0002.tiff Comparison of charged residues at the periplasmic binding cavity of MexB and MexY.

Fig. S3 mbio.03826-24-s0003.jpg MD simulation of the MexY trimer.

Fig. S4 mbio.03826-24-s0004.jpg Putative proton transfer pathway of MexY.

Supplemental Information mbio.03826-24-s0005.docx Legends for supplemental files.

Table S1 mbio.03826-24-s0006.pdf MexY cryo-EM data collection, refinement statistics, and protomer's state assignment.

Table S2 mbio.03826-24-s0007.pdf Docking of drugs to the MexY trimer.

Movie S1 mbio.03826-24-s0008.mov First eigenvector of the MD simulation trajectory.

Movie S2 mbio.03826-24-s0009.mov Second eigenvector of the MD simulation trajectory.

Movie S3 mbio.03826-24-s0010.mp4 Str export via the MexY tunnel connecting the ceiling of the central cavity and the bottom of the funnel.

ASM does not own the copyrights to Supplemental Material that may be linked to, or accessed through, an article. The authors have granted ASM a non-exclusive, world-wide license to publish the Supplemental Material files. Please contact the corresponding author directly for reuse.
