# Supplementary figures and images for: Cryo-EM structure of the Pseudomonas aeruginosa MexY multidrug efflux pump
Source: mBio. 2025 Mar 5;16(4):e03826-24. doi: 10.1128/mbio.03826-24 (PMC11980583; doi:10.1128/mbio.03826-24)

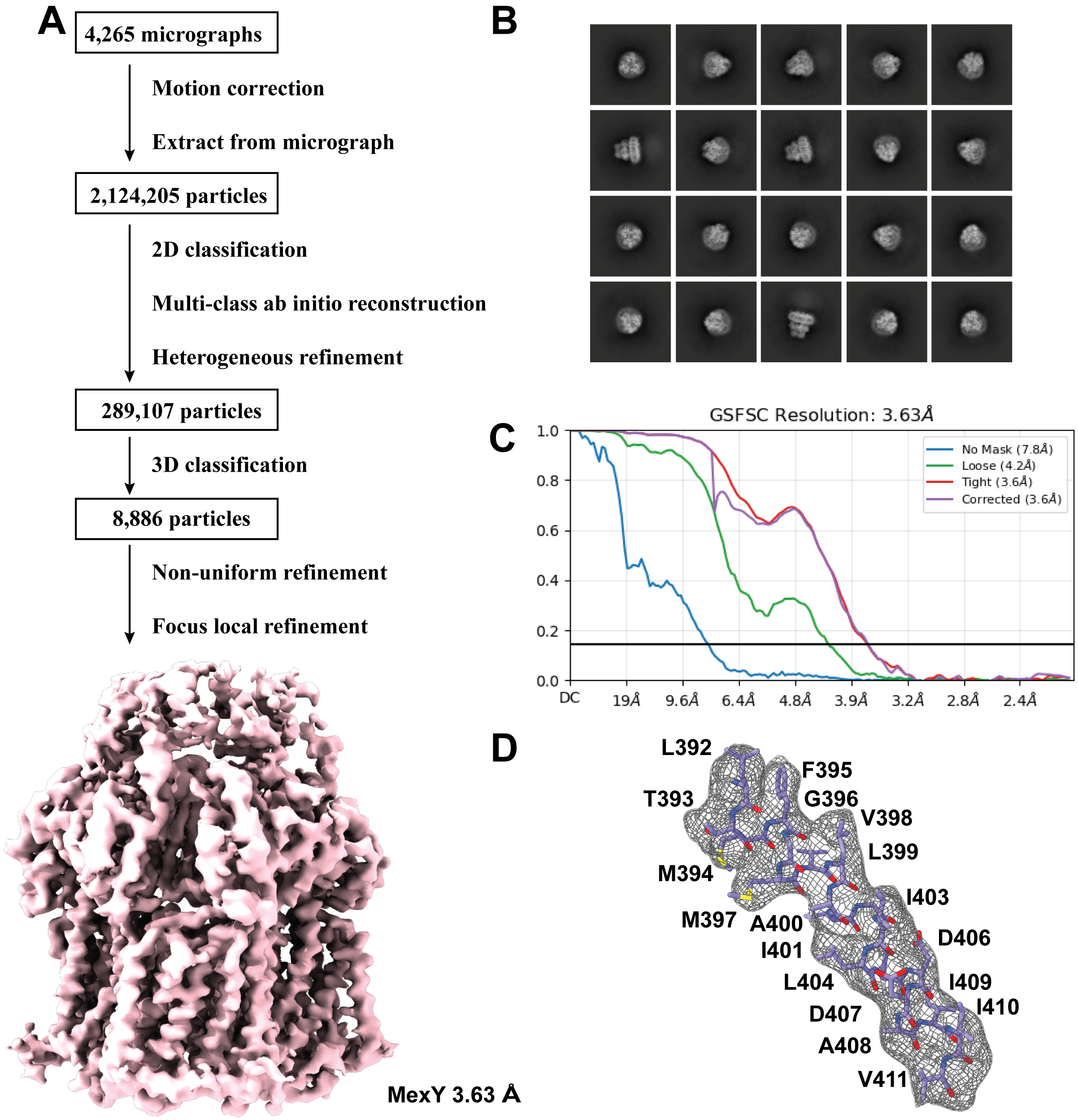

Supplement: Fig. S1 — Cryo-EM structure of the MexY efflux pump. [file mbio.03826-24-s0001.tiff]

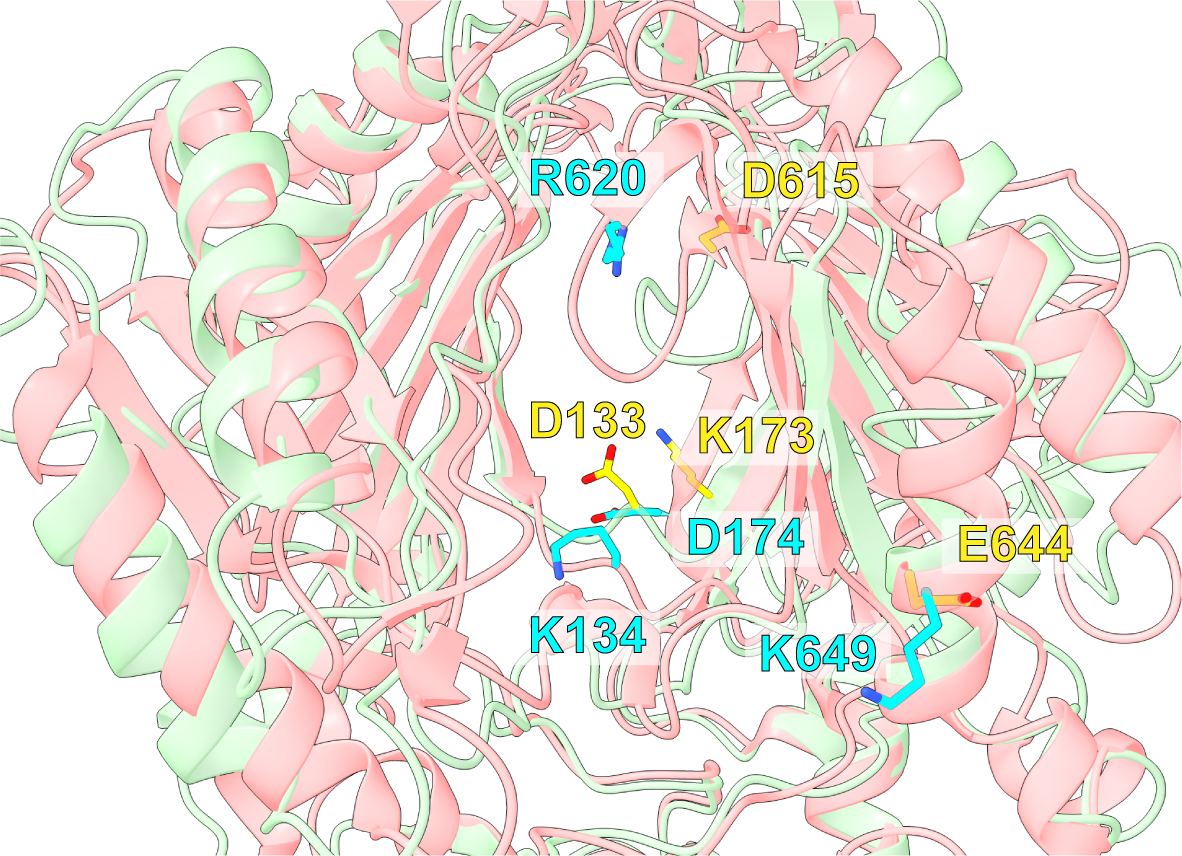

Supplement: Fig. S2 — Comparison of charged residues at the periplasmic binding cavity of MexB and MexY. [file mbio.03826-24-s0002.tiff]

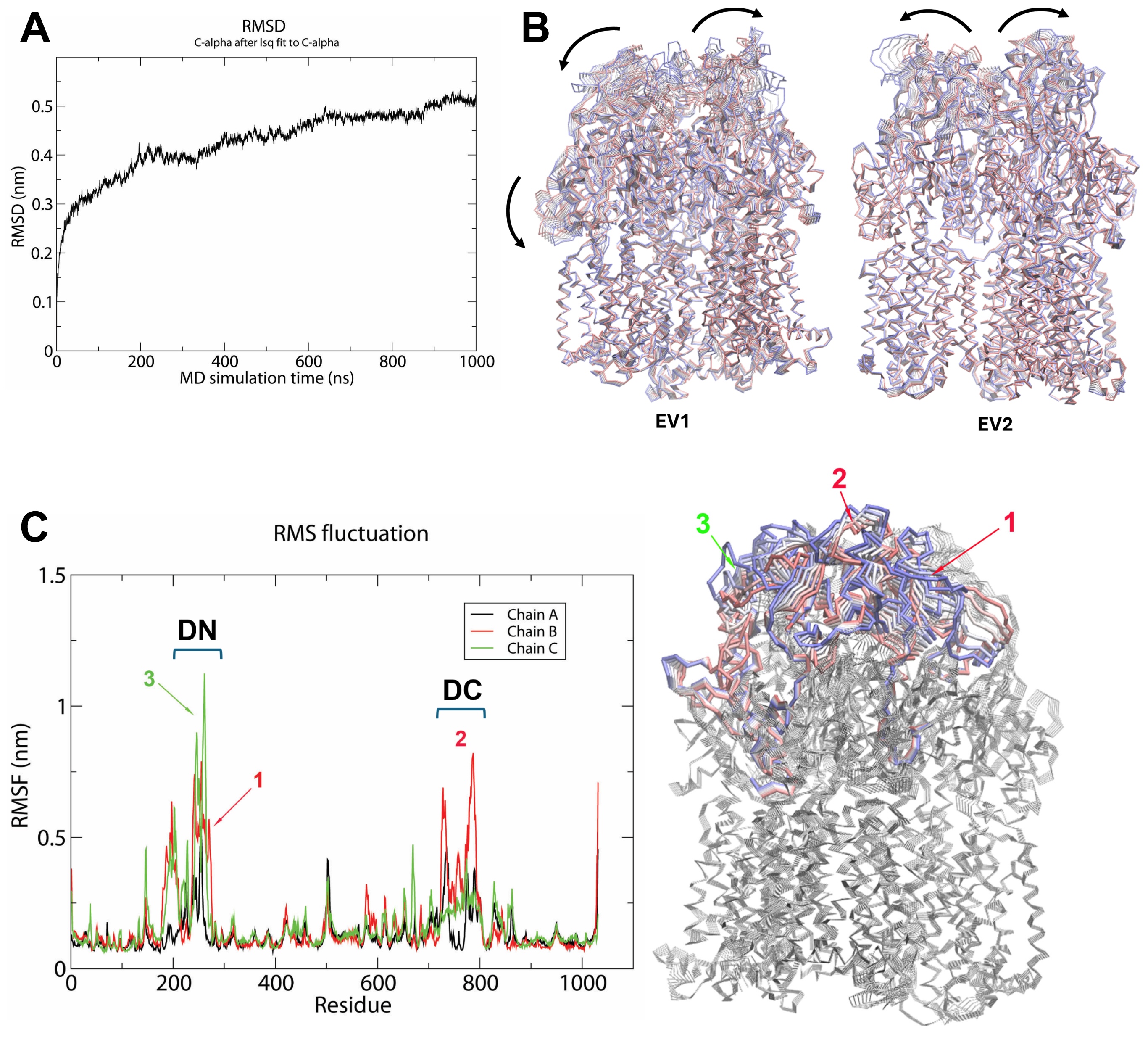

Supplement: Fig. S3 — MD simulation of the MexY trimer. [file mbio.03826-24-s0003.jpg]

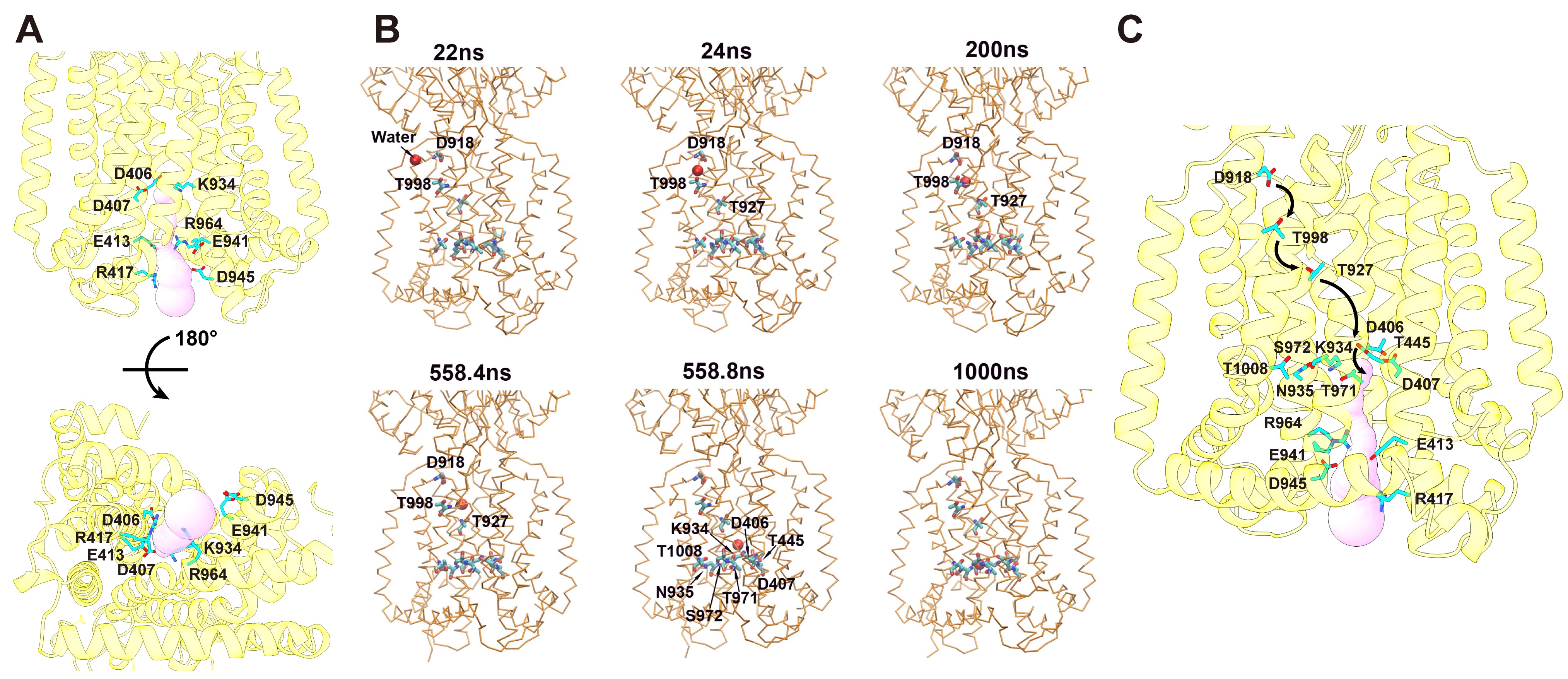

Supplement: Fig. S4 — Putative proton transfer pathway of MexY. [file mbio.03826-24-s0004.jpg]
