# Supplementary material for: Cryo-EM structure of the Pseudomonas aeruginosa MexY multidrug efflux pump
Source: mBio. 2025 Mar 5;16(4):e03826-24. doi: 10.1128/mbio.03826-24 (PMC11980583; doi:10.1128/mbio.03826-24)
Supplement: Supplemental Information — Legends for supplemental files. [file mbio.03826-24-s0005.docx]

**Supplementary Information**

**Supplemental Figures, Tables and Movies**

**Fig. S1. Cryo-EM structure of the MexY efflux pump.** (A) Data processing workflow of trimeric MexY. Side view of the cryo-EM density map of trimeric MexY is shown. (B) Representative 2D classes of trimeric MexY. (C) Gold-Standard Fourier shell correlation (GS-FSC) curve. (D) Local cryo-EM density map of MexY.

**Fig. S2. Comparison of charged residues at the periplasmic binding cavity of MexB and MexY.** The secondary structural elements of MexB and MexY are colored pink and green, respectively. The MexB charged amino acids K134, D179, R620 and K649 are colored cyan. The MexY charged amino acids D133, K173, D615 and E644 are colored yellow. These charged residues are found to switch from positive to negative or vice versa when compared with these two proteins.

**Fig. S3. MD simulation of the MexY trimer.** (A) The RMSD (root mean square deviation) of Cα atoms are based on the MD simulation trajectories. This is a 1 μs simulation. (B) The first and second eigenvectors of trimeric MexY from PCA. Black arrows show the rigid-body motions of the periplasmic domain. Six structures from each eigenvector were overlaid showing these rigid-body motions (blue to red). (C) RMSF (root mean square fluctuation) of Cα atoms of trimeric MexY. The calculations indicate that residues forming subdomains DN and DC of the docking region are quite flexible.

**Fig. S4. Putative proton transfer pathway of MexY.** (A) Formation of a funnel-shape tunnel (pink) below the conserved triad made up of the charged residues D406, D407 and K934. The figure also includes charged residues, E413, R417, E941, D945 and R964, lining the wall of this tunnel. (B) Targeted MD simulations of MexY. This figure depicts snapshots of residues participating in the putative proton-relay network of MexY at (A) 22.0, 24.0, 200.0, 558.4, 558.8 and 1000 ns. The simulations indicate that residues D406, D407, T445, D918, T927, K934, N935, T971, S972, T998 and T1008 form a proton-relay network to facilitate the transfer of proton across the membrane. (C) Proposed pathway of proton transfer in the MexY efflux pump. The important charged and polar residues participate in proton transfer are highlighted with cyan sticks. The water channel is colored pink.

**Table S1.** MexY cryo-EM data collection, refinement statistics, and protomer’s state assignment.

**Table S2.** Docking of drugs to the MexY trimer.

**Supplementary Movie 1.** First eigenvector of the MD simulation trajectory.

**Supplementary Movie 2.** Second eigenvector of the MD simulation trajectory.

**Supplementary Movie 3.** Str export via the MexY tunnel connecting the ceiling of the central cavity and the bottom of the funnel.
