# Supplementary material for: Cryo-EM structure of the Pseudomonas aeruginosa MexY multidrug efflux pump
Source: mBio. 2025 Mar 5;16(4):e03826-24. doi: 10.1128/mbio.03826-24 (PMC11980583; doi:10.1128/mbio.03826-24)
Supplement: Table S1 — MexY cryo-EM data collection, refinement statistics, and protomer's state assignment. [file mbio.03826-24-s0006.pdf]

Table S1A. MexY cryo-EM data collection and refinement statistics.	
**Data collection**	**MexY-Apo**
Magnification	81,000
Voltage (kV)	300
Electron Microscope	Krios-GIF-K3
Defocus (um)	-0.8 to -1.5
Energy filter width (eV)	20
Pixel size (Å)	1.07 (0.535)
Total dose (e^-/ Å^2)	37.6
Number of frames	38
Number of micrographs	4,265
Initial particle images (no.)	2,124,205
**Refinement**	
Total Particles (no.)	8,886
GS-FSC Resolution (0.143, Å)	3.63
<u>Model composition</u>	
Chains	3
Protein residues	3,032
Ligand	0
<u>r.m.s.d.</u>	
Bond lengths (Å)	0.003
Bond angles (°)	0.573
**Validation**	
MolProbity score	2.26
Clash score	14.84
<u>Ramachandran plot</u>	
Favored (%)	96.22
Allowed (%)	3.75
Disallowed (%)	0.03
CC Mask	0.74

Table S1B. MexY protomer's state assignment.

Protomer	Cleft State	Exit site distance, D124 to Y752	Hydrogen bonded distance, K934 to				Protomer Assignment
			D406 (Å)	D407 (Å)	N935 (Å)	T971 (Å)	
MexY, A	Closed	8.1	2.8	2.8	-	-	Resting
MexY, B	Closed	21.3	-	-	2.8	3.0	Extrusion
MexY, C	Open	10.0	2.7	3.0	-	-	Binding

Protomer states were defined using three criteria. (1) State of the periplasmic cleft between PC1 and PC2 (open or closed). (2) Size of the exit site of the extrusion tunnel measured by the distance between the Cα atoms of D124 and Y752, which form the substrate exit site. (3) Hydrogen-bonded distance between the "proton sweeper" K934 and other residues within the proton-relay network (D406, D407, N935 or T971). Only distances of 3.20 Å and under are noted.
