# Supplementary material for: Cryo-EM structure of the Pseudomonas aeruginosa MexY multidrug efflux pump
Source: mBio. 2025 Mar 5;16(4):e03826-24. doi: 10.1128/mbio.03826-24 (PMC11980583; doi:10.1128/mbio.03826-24)
Supplement: Table S2 — Docking of drugs to the MexY trimer. [file mbio.03826-24-s0007.pdf]

**Table S2. Docking of drugs to the MexY trimer.**			
Protomer	ligand	Binding site	Binding energy
Binding protomer			
	tigecycline	Exit/Distal site	-8.1 kcal/mol
	erythromycin	Exit/Distal site	-8.1 kcal/mol
	cefpirome	Exit/Distal site	-7.4 kcal/mol
	amikacin	Exit/Distal site	-7.6 kcal/mol
	streptomycin	Exit/Distal site	-7.3 kcal/mol
Binding protomer			
	tigecycline	Entrance/Proximal site	-10.1 kcal/mol
	erythromycin	Entrance/Proximal site	-6.4 kcal/mol
	cefpirome	Entrance/Proximal site	-6.8 kcal/mol
	amikacin	Entrance/Proximal site	-8.2 kcal/mol
	streptomycin	Entrance/Proximal site	-8.1 kcal/mol
Extrusion protomer			
	tigecycline	Exit/Distal site	-12.1 kcal/mol
	erythromycin	Exit/Distal site	-6.1 kcal/mol
	cefpirome	Exit/Distal site	-6.2 kcal/mol
	amikacin	Exit/Distal site	-7.6 kcal/mol
	streptomycin	Exit/Distal site	-6.3 kcal/mol
Extrusion protomer			
	tigecycline	Central cavity ceiling site	-5.6 kcal/mol
	erythromycin	Central cavity ceiling site	-6.0 kcal/mol
	cefpirome	Central cavity ceiling site	-7.9 kcal/mol
	amikacin	Central cavity ceiling site	-7.6 kcal/mol
	streptomycin	Central cavity ceiling site	-5.9 kcal/mol
